# Artificial Intelligence in the Assessment of Heart Rate Variability as an Instrument to Understand the Connection Between Psychologic and Psychiatric Conditions and the Heart

**DOI:** 10.3390/bioengineering13050554

**Published:** 2026-05-14

**Authors:** Simon W. Rabkin

**Affiliations:** Division of Cardiology, Department of Medicine, University of British Columbia, 9th Floor 2775 Laurel St., Vancouver, BC V5Z 1M9, Canada; simon.rabkin@ubc.ca; Tel.: +1-(604)-875-5847; Fax: +1-(604)-875-5849

**Keywords:** artificial intelligence, machine learning, mental stress, anxiety disorders, panic disorder, depression, schizophrenia

## Abstract

Heart rate variability (HRV) refers to variations in the time intervals between consecutive heart beats. Changes in HRV reflect changes in either sympathetic or decreased parasympathetic tone that can originate in the brain. This brain–heart connection has led to the proposal that HRV may have utility in the diagnosis of psychiatric conditions and/or be a predictor of the response to psychiatric medications. There have been attempts to improve the correlation between HRV and psychological and psychiatric conditions by using artificial intelligence or specific machine learning algorithms. The objective of this review is to synthesize data on the use of machine learning to improve accuracy in differentiating psychological conditions such as mental stress, as well as distinguishing persons with anxiety disorders, panic disorders, major depression disorders and schizophrenia from health subjects. Reported accuracies for the identification of mental stress vary from 42 to 94%, while accuracies for anxiety vary from 67 to 98%, panic disorders from 71 to 93% and depression from 71 to 95%. The ability of HRV to differentiate different psychological or psychiatric conditions from each other requires more investigation. The ‘best’ machine learning algorithm varied between studies, with some reporting the k-nearest neighbor algorithm, support vector machine, random forest, or neural networks to be the best. A number of studies combined HRV with other variables such as respiration, EEG, or electromyography to obtain a composite index, but in doing so obscured the independent contribution of HRV. In summary, HRV has shown promise in detecting abnormalities in a range of psychological and psychiatric conditions. The use of machine learning algorithms improves diagnostic accuracy.

## 1. Introduction

Heart rate monitoring has received increasing attention in the assessment of health and fitness, in part due to the growing availability of wearable sensor devices that enable the continuous and accessible measurement of heart rate [1,2]. Persistently slow, elevated, or irregular heart rates may indicate underlying cardiac abnormalities of clinical concern. Heart rate variability (HRV), defined as the beat-to-beat variation in heart rate over time, provides a non-invasive measure of autonomic regulation, reflecting sympathetic and parasympathetic influences on cardiac function [3,4]. HRV is altered in states of psychological stress and in individuals with major psychiatric conditions, including panic disorder, major depressive disorder, and schizophrenia [5,6].

Emerging evidence suggests that HRV may have diagnostic utility in these psychiatric conditions [7]. Furthermore, HRV has been proposed as a potential predictor of treatment response to antidepressant medications, with higher HRV associated with more favorable outcomes and lower HRV linked to poorer responses [8]. This raises the question of how to enhance the utility of HRV in the assessment of psychological and psychiatric disorders.

**Figure 1:**
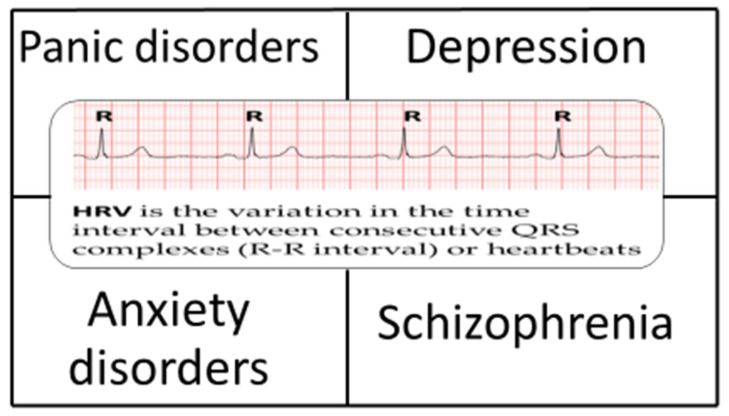
The key mental health conditions that have been linked to heart rate variability.

Artificial intelligence (AI), particularly machine learning (ML), has increasingly been applied to the analysis of prolonged electrocardiographic (ECG) recordings, demonstrating improved performance [9], similar to its established value in 12-lead ECG interpretation [10,11]. In this context, ML-based approaches have been explored to leverage HRV data more effectively. A systematic evaluation of the ability of ML methods to utilize HRV for the assessment of psychological and psychiatric conditions is therefore warranted and forms the focus of this analysis (Figure 1).

## 2. Heart Rate Variability (HRV)

Fluctuations in heart rate, labeled heart rate variability (HRV), are not only a reflection of alterations in autonomic tone [3,4] but may be an indicator of cardiovascular disease [12,13]. HRV has been assessed or measured utilizing either time domain methods or frequency domain methods [12,13]. The *time domain* methods measure the heart rate at a given time point or the intervals between successive normal complexes. Time domain measurements include: pNN50 (percentage of adjacent NN (RR) intervals that differ from each other by more than 50 ms), rms-SD (obtained by first calculating each successive time difference between heartbeats in ms; then, each of the values is squared and the result is averaged before the square root of the total is obtained), SDNN (standard deviation of the inter-beat interval of normal sinus beats), SDANN (standard deviation of the average NN intervals for each 5 min segment of a 24 h HRV recording), and Tri or the triangular index (baseline width of a histogram displaying NN intervals). The *frequency domain* methods rely on the power spectral density, which provides information on the distribution of variance as a function of frequency [12,13]. There is a strong correlation between time domain and frequency domain variables when measured over prolonged time frames [13]. There is a circadian variation in HRV [14].

This research question aims to determine the relationship between heart rate variability after artificial intelligence or machine learning modeling and mental stress, anxiety disorders, panic attacks, depression, and schizophrenia. This systematic review was conducted according to the Preferred Reporting Items for Systematic Reviews and Meta-Analyses (PRISMA) guidelines. The review protocol was not previously published. A literature search was conducted across MEDLINE from database inception to 31 October 2025. The following inclusion criteria were used: adult (age ≥ 18) persons undergoing mental stress or diagnosed with anxiety disorders, panic disorders, depression or schizophrenia. Exclusion criteria included non-human studies, pediatric age groups, and editorials, commentaries, conference abstracts, reviews, or non-English studies to ensure the methods and results could be reviewed in detail (see Supplementary Materials).

## 3. Artificial Intelligence/Machine Learning

Data analytics, approached under the rubric of machine learning, have become part of the ‘tool box’ to discriminate a condition from the absence of that condition [10]. There are a number of approaches and each has advantageous and limitations. A brief outline of some of these is presented as follows:

**K-nearest neighbor** (**KNN**) The k-nearest neighbors (KNN) algorithm is a non-parametric, supervised learning classifier, which uses proximity to classify individual data points that form a group, defined based on their proximity to each other or ‘K’ closest neighbors in the feature space, using distance metrics like Euclidean distance [15]. This approach has been useful in clinical medicine [16].
**Support vector machine (SVM)** is a supervised machine learning algorithm used for classification and regression. It attempts to find the best “hyperplane” (decision boundary) to separate data into categories, maximizing the margin (distance) to the closest points (support vectors) for robust, accurate predictions, and is useful in a variety of clinical situations [16,17,18].
**Logistic regression (LR)** is used to obtain the odds ratio in the presence of more than one explanatory variable in order to identify the contribution of each variable or the odds of the observed event of interest [19].
**Linear discriminant analysis (LDA)** separates multiple classes with multiple features through data dimensionality reduction and is especially useful in separating or differentiating two groups [20].
**Naïve Bayes** constructs a family of supervised machine learning algorithms that use Bayes’ Theorem for classification and assumes that the features are conditionally independent [21].
**Decision tree (DT)** is a flow chart-like model that maps out possible actions in a hierarchical manner that resembles a tree [22]. **Extremely Randomized Trees Classifier** (**ERTC**) randomizes both attribute and cut-point choice while splitting a tree node [23].
**Random forest (RF)** is an ensemble machine learning method that builds a number of decision trees in the training set and combines their predictions, i.e., construct a diverse group of models that collectively out preform a single tree [24].
**Gradient boosting machine (GBM)** is an ensemble learning algorithm that produces accurate predictions by combining multiple decision trees into a single model. It builds accurate models by sequentially combining many simple models (usually decision trees) to minimize prediction mistakes for complex regression and classification tasks [25,26].
**LightGBM Gradient Boosting Decision Tree** (**GBDT)** utilizes a Gradient-based One-Side Sampling (**GOSS**) and Exclusive Feature Bundling (**EFB**) that uses tree-based learning algorithms, providing an approach that excels at the classification, regression and ranking of data.kml.,
**eXtreme Gradient Boosting** (XGBM) implements the gradient boosting tree algorithm [27]. It is an open-source machine learning library known for its speed, accuracy, and scalability. It has been used in a wide variety of medical applications [28].
**Fuzzy logic models** use mathematical fuzzy logic to deal with uncertainty and imprecision [29].
**Neural networks** functions use layers of interconnected nodes to learn patterns from data in order to recognize images and understand language. Recurrent neural networks (**RNNs**) have been useful for a variety of health care issues [30,31]. A long short-term memory architecture (**LSTM**) is a special type of neural network designed to learn and remember information over long sequences of data.
**Multilayer perceptron** (**MLP**) is a type of neural network with connected nodes organized in layers which is adept at handling non-linear data [32].

## 4. Mental Stress

HRV has been known to be altered under mental stress and has been suggested to be a way to detect psychological stress [33,34,35]. A recent review concluded that HRV is a valid measure of the psychological stress response [36]. This has been demonstrated both in acute responses to stress and more sustained levels of stress [37]. Mental stress is associated with changes in HRV consistent with a decrease in parasympathetic nervous system activity and an increase in sympathetic nervous system activity [38]. In a review, low parasympathetic activity, characterized by a decrease in high-frequency power HRV and an increase in low-frequency power HRV, was reported to be the most common factor associated with changes in stress [39]. Based on neuroimaging studies, HRV may be linked to specific cortical regions, such as the ventromedial prefrontal cortex, that are involved in stressful situations [39].

A number of studies have applied AI or ML algorithms to HRV data from mental stress. He et al. studied the response of 26 college students to a series of stressful or non-stressful conditions [40]. Using an SVM architecture in a binary assessment, the presence of stress was identified with a classification accuracy of 76% or, when using a multi-class classification approach, with an accuracy of 79% [40]. The small sample size employed to develop the algorithm is an issue.

Cinaz et al. evaluated the ECG using a 2 min recording, with a small sample of seven subjects given different workloads as the stressor [38]. They employed three classification methods, linear discriminant analysis (LDA), k-nearest neighbor algorithm (KNN), and SVM (with linear kernel), and compared their performance. The best results were obtained with LDA, which yielded a correct classification for six out of the seven subjects. The KNN and the SVM resulted in a correct classification of the mental workload level during office work for five out of the seven subjects [38]. The major limitation of this study was the small sample size and the absence of a second sample to test their algorithm.

Fan et al. studied 20 healthy, young males (mean age of 25 years), collecting EEG and ECG data, which were combined to establish an evaluation model based on SVM [41]. Before the classification, principal component analysis was used to extract the principal elements and decrease the dimension of sample space in order to simplify the calculation. An SVM effective classification model was developed with an accuracy of 80% for mental workload [41]. The small sample size, the combination of EEG and ECG data and the absence of a second sample to test their algorithm are major limitations of this study.

Parent et al. used a generalized linear regression model to analyze data from 18 students (14 men) at a civil aviation school without reported psychological, neurological conditions or cardiovascular disease, who were not taking medication affecting the brain or autonomic functions [42]. They computed RR intervals and used Kubios HRV software (v 2.2) to obtain 22 ECG features. Nine features were obtained using time domain analysis (e.g., mean heart rate, RMSSD) and 13 were obtained using frequency domain analysis (e.g., LF and HF power). Their model achieved an average accuracy of 42% for HRV [42]. The relatively low accuracy of the ECG was attributed to the nature of the stressor, which was only of moderate emotional intensity, producing a small increase in heart rate. They postulated that a more intense stress, generating a more marked physiological response, would produce a higher accuracy but they did not test this hypothesis [42].

Giannakakis et al. evaluated 24 persons (age 47 years) of whom 17 were men and collected ECG data that extracted various HRV parameters [43]. Stressors were designed to simulate a range of everyday life conditions (social exposure, stressful event recall, cognitive load, and stressful videos). The most important HRV parameters measured were the ability to discriminate between stress and no-stress conditions. Six different models, the KNN, Generalized Linear Model, Naïve Bayes, LDA, SVM, and RF classifiers, were assessed. The RF approach outperformed all other classification schemes and had an accuracy of 75%. When performance was investigated using pairwise transformed selected features, the best performance reached a classification accuracy of 84% and was obtained using the SVM approach [43]. A second data set was used to test their algorithm.

Iovino et al. collected data from 127 young healthy volunteers (75 females, 52 males; age: 18 years) who wore ECGs and received a non-verbal mental test consisting of a repetitive visualization of randomly generated three-digit numbers that appeared on the ceiling of the room. Four classic ML classifiers—LDA, SVM, KNN and RF—were selected and their performance in discriminating between rest, orthostatic stress and mental stress was evaluated and compared. There were no significant differences between the ML classifiers, and accuracy was reported at around 80% [44].

Castaldo et al. recorded ECG of up to 5 min in duration for 42 healthy persons during a university exam, which served as the stressor compared to resting conditions [45]. LDA, multilayer perception and a C45 decision trees showed a similar accuracy of 94% for different ECGs in the same data set; no training or test data sets were used [45].

Bahameish et al. used three available data sets: their first data set contained 39 individuals and was used to develop the model, which was tested on two other data sets with a combined total of 40 people [46]. Participants were placed under conditions of cognitive stress, and paced breathing and HRV was calculated for 5 min (300 s) of data from a photo plethysmograph sensor. Six different ML algorithms were evaluated: logistic regression, decision trees (DT), k-nearest neighbors, Naive Bayes, random forest, and support vector machine. There were differences in accuracy between the different models in the primary data set. The highest F1 score was attained by the random forest model (Table 1).

In the secondary data set (that was not used to develop the ML algorithm), the random forest model showed a considerably lower F1 score (Table 2).

These data highlight the need to use secondary data to examine the value of the algorithm developed from the first data set.

Lei et al. studied twenty inexperienced scaffolding workers who were young men; ECG signals were collected while the men were working at three different heights, corresponding to low, medium, and high levels of mental stress [47]. Machine learning algorithms, including SVM, KNN, LDA and RF, were applied for model development. HRV features obtained good prediction accuracy. The classification accuracy was up to 85% between low and medium stress levels, 93% when differentiating low and high stress levels using KNN, and 88% for classifying medium and high stress levels. There was no test group, only a training group with a small number of young men aged 19 to 23 years [47].

Lee et al. studied 74 third-year police officers without heart disease (mainly young men) who were subjected to a Tier Social Stress Test and horror movie viewing while wearing a heart rate monitor attached to a chest strap [48]. Using short-term (5 min) and ultrashort-term (less than 5 min) calculations of HRV, the SVM accuracy was 87 and 91%, respectively. There was no training or test evaluation [48].

Hwang et al. collected ECG data from 13 young students at Kwangwoon University in Korea and 9 at KU Leuven University in Belgium, who were subjected to different stressors including mental arithmetic test, the Stroop color–word test, a scripted interview, a visual stimuli test and the cold pressor test [49]. Ten conventional machine learning algorithms were used as classifiers: the highest classification for one data set was 73%, obtained using an RF algorithm and the highest classification for the other was 67%, obtained using a Multilayer Perceptron (MLP) algorithm [49].

A number of studies are mentioned briefly because they assessed multiple assessment techniques together, precluding an accurate evaluation of HRV alone. Pourmohammadi et al. applied a cluster analysis to ECG and EMG data collected from 34 university students (11 males and 23 females) who were subjected to mental stress. Mental arithmetic at three levels of difficulty and the Stroop color–word tasks were used [50]. A fuzzy-based model was employed, which used the combined ECG and electromyogram signals to achieve an average stress classification accuracy across all subjects for two and three levels of stress and achieved an accuracy of 97% and 76%, respectively [50]. The combination of EEG and ECG data precludes assessment of HRV alone.

Singh et al. used Photo plethysmography (PPG) and Galvanic Skin Response (GSR) to assess physiologic reactions to stress in persons driving a car, and HRV was calculated from the PPG [35]. They reported that Layer Recurrent Neural Networks was the most optimal method for stress level detection. This evaluation achieved an average precision of 89.2%, sensitivity of 88.8% and specificity of 94.9% when tested over 19 automotive drivers [35]. Betti et al. collected data from 15 healthy individuals using multiple sensors and assessed the response to stress by evaluating a combination of ECG and electro-dermal and brain activity via SVM analysis, and reported an accuracy of 86% [51]. Xu et al. collected Galvanic Skin Response (GSR), electromyography (EMG), heart rate (HR), and EEG measurements on 39 subjects, with 21 belonging to the physical task group and 18 to the cognitive task group [52]. They used KNN and found that it significantly improved the accuracy in the detection of stress as compared to traditional methods without clustering [52]. Li and Liu analyzed data from the Machine Learning Repository, hosted by the University of California, from 15 human participants who experienced baseline, amused, and stressed conditions [53].The sensors included an ECG, electrodermal activity sensor, electromyography sensor, skin temperature sensor, respiratory rate sensor, and three-axis accelerometer. They developed two deep neural networks. The deep convolutional neural network achieved 99.8% and 99.6% accuracy rates for binary and three-class classification, respectively. The deep multilayer perceptron neural network achieved 99.7% and 98.4% accuracy rates for binary and three-class classification, respectively [53]. The small sample size and multiple inputs limit the ability to derive conclusions on the contribution of HRV to assess psychological stress.

Can et al. combined physiological data for heart activity, skin conductance and accelerometer signals, obtained from 21 students attending an algorithmic programming contest summer camp, and compared a high-stress cognitive load (lecture) with relaxed activities using different machine learning methods [54]. They reported 90% accuracy for the single data set [54]. Gedam et al. utilized a data set of 200 participants who were monitored during four different stressors [55]. Nine ML algorithms were investigated for both multivariate and univariate features. The physiological data was collected using ECG, GSR, and ST sensors. The findings reveal that the suggested model detects mental stress with an accuracy of 96%, with the XGBoost method outperforming other algorithms in multivariate analysis. Univariate feature analysis found that XGBoost regularly demonstrated good accuracy in detecting mental stress. Additionally, benchmark data set validation (SWELL-KW, WESAD) confirmed the model’s robustness, with accuracies of 92% and 94% respectively [55]. The isolated accuracy of HRV could not be assessed.

In summary, ML algorithms utilizing HRV data provide a good level of accuracy in identifying an individual’s perception of the presence of mental stress, but the literature does not always distinguish HRV from other physiologic measurements, such as EEG, electromyography and Galvanic Skin Response. In addition, most studies had small sample sizes, often with a young population, and many did not use training and test data sets to evaluate their ML models. The reported accuracy varies widely, reflecting the population studied, the absence of training and test groups and the AI/ML model constructed.

## 5. Anxiety Disorders

From a global perspective, anxiety disorders are the most common type of mental illness [56]. As anxiety and depression frequently coexist [57], meaning that it can be challenging to differentiate these conditions, the use of wearable devices in conjunction with combination of AI technology has been proposed for the detection and prediction of anxiety disorders [58,59]. Autonomic abnormalities have been identified in persons with anxiety disorders [60]. Interestingly, apparently healthy individuals with high test scores for traits related to anxiety, that represents an important risk factor for anxiety disorder, have manifestation of autonomic dysfunction [61].

Alkurdi et al. studied 15 individuals (20% female) and recorded ECG, skin conductance, EMG, respiration, skin temperature, and three-axis acceleration (ACC), from wearable devices [62]. Feature-based models, particularly XGBoost and Decision Trees, demonstrated considerable resilience, maintaining higher accuracy and reliability [62]. The incorporation of so many different input modalities limits the assessment of HRV alone. Indeed, a literature review of multiple input variables concluded that the EEG was the best performer, although accurate results were obtained with heart rate monitoring [63]. Random forest and support vector machines led to good results and neural networks provided good accuracy [63]. This review also comments on the effective combinations of modalities and the success of different models for detecting anxiety [63].

Gu and Hu examined the HRV data set from the Amigos data set (a data set for multimodal research on affect, personality traits and mood in individuals and groups) that assessed 40 persons who watched 16 short emotional videos and 4 long videos [64]. Their SVM model had an accuracy of 67%, an LSTM model had an accuracy of 73% and combining the two obtained an accuracy of 86% [64].

Li et al. studied 845 university students between the ages of 18 and 22 years who reported good health [65]. Participants completed self-assessment scales for anxiety and depression (Self-Rating Anxiety Scale (SAS) and the Patient Health Questionnaire-9 (PHQ-9). HRV data were collected during exercise and for a 5 min period post-exercise. The multilayer perceptron neural network model, which included several branches with identical configurations, was employed for data processing. The accuracy of the model in predicting anxiety levels was 89% for no anxiety, 84% for mild anxiety, and 79% for moderate to severe anxiety [65].

Handouzi et al. studied 45 individuals with slight to moderate anxiety according to the LSAS scale and individuals diagnosed with social phobia [66]. Heart rate variability was extracted from a sensor that measures fluctuations in blood volume within arteries and capillaries by emitting infrared light through the tissues [66]. They reported 98% accuracy using an LSTM model [66]. There is concern about the HRV measured by this method compared to the more accurate ECG assessment.

One study that did not use AI/ML to isolate the relationship between HRV and anxiety is useful to review. Bilgin et al. investigated the relationship between HRV frequency sub-bands and anxiety tests in patients with fibromyalgia (56 persons diagnosed according to the American College of Rheumatology criteria and healthy controls (*n* = 34)) [67]. HRV sub-bands were obtained from the ECG signals using Wavelet Packet Transform. The sub-bands and anxiety tests scores were analyzed and compared using multilayer perceptron neural networks (MLPNN). They found that the HRV high-frequency (HF) sub-band in the range of 0.15235 Hz to 0.40235 Hz was correlated with the Beck Anxiety Inventory (BAI), and another HRV HF sub-band, with a frequency range of 0.15235 Hz to 0.28907 Hz, was correlated with doctor-rated Hamilton Anxiety Inventory (HAM-A) scores. The overall accuracy was 91.1% for HAM-A and 90% for BAI with MLPNN analysis [67].

In summary, there is not an abundance of data on AI to improve the diagnostic classification ability of HRV data regarding anxiety disorders but the data are encouraging. Most studies did not compare the accuracy of their AI model with that of HRV prior to application of the AI model.

Anxiety and depression frequently coexist [57], so it can be challenging differentiating these conditions. AI has been used to assess self-reported questionnaire data for predicting the presence of depression and anxiety [68]. The use of HRV should improve the results from self-administered questionnaire data and this should be tested in future research.

AI programs have been developed and proposed to manage anxiety disorders to deal with the inadequate availability of face-to face psychotherapy [69]. An intriguing possibility is the utilization of AI algorithms to couple the diagnosis and treatment of anxiety disorders.

## 6. Panic Disorders

Panic disorder is an anxiety disorder associated with unexpected panic attacks, which are characterized by sudden surges of intense fear along with physical symptoms of palpitations, dyspnea and dizziness, accompanied by behavior changes such as avoidance strategies [70]. It has been suggested that brain amygdala hyperactivation is involved, with a major role for serotonergic, noradrenergic and glutamatergic neurotransmitters in its pathophysiology [71].

Tsai et al. collected data on patients with panic disorders, from a single general hospital, by using wearable devices recording heart rate [72]. HRV was calculated from the natural logarithm of the standard error of the time domain normal-to-normal RR interval, derived from the 5 min continuous heart rate measurement [72]. Accuracy was 93% for LSTM and 91% for RNN [72].

Na et al. studied 60 patients with panic disorder and 61 patients with other anxiety disorders (aged between 20 and 65 years) [73]. Twenty-four percent had comorbid psychiatric disorders, mainly depression. The HRV was assessed in high- and low-frequency domains; the 0.15–0.4 Hz area is referred to as the high-frequency domain and the 0.04–0.15 Hz area as the low-frequency domain. Five algorithms were used: logistic regression (LR), artificial neural network (ANN), gradient boosting machine (GBM), random forest (RF), and support vector machine (SVM). The LR showed the best accuracy (78.4%), followed by ANN (73.0%), SVM (73.0%), GBM (67.6%), and finally RF (64.9%). LR also showed good performance in other measures, such as F_1_-score (79.0%), specificity (73.7%), sensitivity (83.3%), and Matthews correlation coefficient (0.572) [73]. The absence of a control group without mental health disorders is unfortunate, as this approach cannot be used as a diagnostic tool. They did not separate the kinds of anxiety disorders, for example, social phobias, which have discrete neurobiological substrates and clinical presentations [73].

Oh et al. examined ECG signals retrieved from a large-scale multi-institutional data set provided by Shaoxing People’s Hospital and Ningbo First Hospital [74]. HRV was calculated using the root mean square of successive differences (RMSSD). The RF model identified panic disorders with an accuracy of 71.4%, precision of 83.7%, recall of 70.6%, and F1 score of 76.6% [74].

In summary, ML algorithms provide a reasonably good approach to identifying individuals with panic disorders.

A study that found a relationship between HRV and panic disorders but did not use ML algorithms is useful to consider. Hong et al. evaluated 110 outpatients diagnosed with panic disorder who visited their outpatient facility, had HRV measurements, and completed the Panic Disorder Severity Scale-Self Report, Beck Depression Inventory (BDI-II), and Insomnia Severity Index [75]. They found that the ratio of low-frequency/high-frequency (LF/HF) HRV parameters was reduced in patients with panic disorders who had depression. Significant correlations were found between depressive symptoms and SDNN, very-low-frequency (VLF), LF, and HF. They concluded that HRV indices may be useful markers for detecting depressive symptoms in patients with panic disorder [75].

## 7. Depression

It has been noted that it can be challenging for mental health professionals to diagnose certain mental health conditions, as they may be affected by patients’ words and speech; therefore, the use of biological factors coupled with machine algorithms, which are emotionless, may improve diagnosis [68]. HRV is a biological factor that has been found to be altered in individuals with major depressive illnesses [76,77,78,79,80,81,82,83]. The data suggest significantly lower sympathetic dominance in subjects with major depressive disorder (MDD) compared to control subjects during instances of stress [83]. Meta-analyses indicated that adults with depression exhibit lower resting HRV indices, including the standard deviation of average normal–normal intervals (SDNN), root mean square of successive differences (RMSSD), proportion of normal complexes differing by more than 50 ms (PNN50), and low-frequency (LF) and high-frequency (HF) parameters compared to healthy controls [5,84]. HRV has been proposed as a biomarker of major depression [7,8,78].

HRV has also been advanced as a potential biomarker for predicting response to antidepressant medications [8]. A meta-analyses based on 18 articles that consisted of 673 depressed participants and 407 healthy comparison participants found that participants with depression had a significantly lower HRV than healthy control subjects [78]. In addition, the severity of the depression was negatively correlated with HRV [78]. This association is likely independent of the medications used to treat MDD, as only tricyclic medication decreased HRV, while serotonin reuptake inhibitors, mirtazapine, and nefazodone had no significant impact on HRV despite patient response to treatment [78].

Several studies used ML to examine HRV in depression. Kobayashi et al. evaluated seven psychiatric patients (three males and four females, mean age 47 years), of whom five had MDD and two had somatoform disorder (SD), as well as sixteen healthy subjects (seven males and nine females, mean age 42 years) [85]. The R-R interval time series was calculated from the time difference in R-wave peak from the ECG. Two kinds of stresses were administered—paced deep breathing and the mental task of verbalizing random numbers between 0 and 9 at intervals of 1 s with the assistance of an electric beat sound. A pause and a rest period were placed between stress times. An SVM was built using several HRV indices to classify subjects as healthy subjects or psychiatric patients. Estimated parasympathetic nerve activity was increased in patients with MDD compared to controls during the mental task. The reported sensitivity was 71.4%, with a specificity of 93.8% and accuracy of 87.0% [85]. The small sample size of persons with depression and the lack of an assessment on a second population, distinct from the one from which the SVM was constructed, are obvious critiques of their model.

Zhang et al. collected HRV data from 10 patients with depression and an equal number of healthy controls who wore an ECG monitor while viewing a 13 min multimodal affective contents stimulus that aimed to induce a variety of emotions [86]. HRV activity was transformed and analyzed with a neuro-fuzzy network model that yielded a reported accuracy rate of 95% [86].

Sun et al. applied a power spectral analysis to R–R interval data from HRV before, during, and after mental task conditions [87]. The mental task condition—random number generation—was performed in 44 drug-naïve patients with MDD and 47 healthy control subjects. Logistic regression analysis was used and performed better than a subjective assessment, achieving a sensitivity and specificity of 80.0 and 79.0%, respectively, and when using their data, accuracy was 79% [87].

Kuang et al. studied 38 women from a hospitalized group (in China) with a mean age of 30 years who were diagnosed with depression by a psychiatrist [88]. The Ewing test was used to perform an evaluation of the autonomic nervous system. The deep breathing and Valsalva states are parasympathetic stimuli, and the standing state applies parasympathetic and sympathetic stimuli. Five HRV features were calculated based on the RR intervals using time domain analysis. Using a Bayesian network algorithm, they reported a 89.5% sensitivity, 84.2% specificity, and 86.84% accuracy [88].

Kim and Lim evaluated 10 patients with depression and 14 healthy controls who were shown meditation and a Funniest Video [89]. From the HRV data, 22 features were extracted and analyzed using a neuro-fuzzy algorithm. Combining the two contents showed the highest mean accuracy, which was 85.4% [89]. The small sample size and lack of a second group were limitations of the study.

Byun et al. collected HRV data from 37 MDD patients and 41 healthy controls during five 5 min experimental phases that consisted of measurements during baseline, a mental stress task, stress recovery, a relaxation task, and relaxation task recovery [90]. Twenty HRV indices were extracted from each phase, and a total of 100 features were used for classification. Using an SVM model, they achieved a 74.4% accuracy, 73% sensitivity, and 75.6% specificity [90].

In a study by Li et al., 845 university students between the ages of 18 and 22 years who reported good health had HRV data collected during exercise and for a 5 min period post-exercise [65]. The multilayer perceptron neural network model, which included several branches with identical configurations, reported an accuracy of 90% for no depression, 84% for mild depression, and 82% for moderate to severe depression [65].

Geng et al. evaluated 80 subjects with a complete polysomnographic (PSG) signal data and extracted HRV data [91]. There were 40 persons with major depression (MDD) and 40 healthy controls (1:1 gender ratio). An SVM and an ERTC model were used. ERTC is an integrated learning technique for classifying data based on the ensemble learning of decision trees, DT, which aggregates the results of multiple de-correlated decision trees collected in a forest to output the classification. Their results show differences between the models (Table 3).

Xia et al. enrolled a total of 165 MDD patients and 60 healthy controls in their study, with each participant completing 24 h Holter electrocardiogram (ECG) monitoring and psychological scale assessments. The circadian rhythm of HRV was quantified using a cosine regression model, and seven typical ML models were employed to distinguish MDD from healthy controls [92] (Table 4).

Yang et al. evaluated data of 465 outpatients that had taken a Depression Assessment Scale and were wearing a plethysmograph to collect HRV, which was measured for 5 min [93]. Logistic regression (LR), support vector machine (SVM), random forest (RF) and eXtreme gradient boosting (XGBoost) algorithm models were used to construct risk prediction models in the training set, and the model performance was verified in the test set. They found that time domain variables (SDNN, SDNN5, pNN50, rmsSD, and SDSD), frequency domain variables (VLF and LF) and nonlinear variables (SD1 and SD2) of HRV were lower in the depression group compared to the non-depression group [93]. The four models were evaluated by area under the receiver operating characteristic curve (ROC), calibration curve, and decision curve analysis (DCA). Furthermore, the SHapley Additive exPlanations (SHAP) method was used to illustrate the effects of the features attributed to the model [93]. In the training set (*n* = 325) and test set (*n* = 140) [93], the area under the curve (AUC) values of the XGBoost model were 0.92 and 0.82 respectively, which were higher than the results of the other three models. Thus, the test set had an AUC of 82% and an F1 score of 79%. The XGBoost model had excellent predictive efficacy and clinical utility. The authors did not present accuracy data to compare their results with other studies [93]. However, they concluded that the HRV-based Boost prediction model had a strong prediction performance and excellent clinical utility [93].

Several studies combined HRV and other factors to identify depression. Saad et al. used a logistic regression with lasso regularization model on data from 1203 polysomnograms from individuals with depression referred to a sleep clinic for the assessment of sleep abnormalities (*n* = 664) and mentally healthy controls (*n* = 529) [94]. The final algorithm was tested on a distinct sample (*n* = 174) to categorize each individual as depressed or not depressed. The resulting categorizations were compared to medical record diagnoses [94]. The algorithm had an overall classification accuracy of 80%, sensitivity of 83% and specificity of 77%. The algorithm remained highly sensitive across subgroups stratified by age, sex, depression severity, comorbid psychiatric illness, cardiovascular disease, and smoking status [94]. However, they ‘integrated multiple features of ECG dynamics including heart rate and heart rate variability, as well as sleep stages scored from the EEG’ [94], which makes it challenging to isolate the role of HRV independent of knowledge of the sleep stage.

Xiao et al. evaluated 55 participants, 23 of whom were depressed patients (condition group) alongside 32 healthy volunteers (control group), who were assessed by the Montgomery–Åsberg Depression Rating Scale (MADRS), but there was little clinical information about these individuals [95]. The data set combines diverse wearable sensor physiological signals, including HRV, electrodermal activity, respiration rate, accelerometer data, which monitors physical activity and movement patterns, and sleep sensors, which monitor sleep stages [95]. Thus, the results cannot be used to assess only HRV and depression. Their AI analytic technique was interesting in that it advanced a dynamic convolutional encoder model based on a Temporal Circular Residual Convolutional Network (DCEM-TCRCN) [95].

In summary, there is robust data, based on large sample sizes, that LM algorithms provide good classification ability in the detection of major depression illness. There is a wide range of reported accuracies, for the reasons cited above.

## 8. Schizophrenia

HRV was found to be altered in schizophrenia [96,97,98,99,100,101,102,103,104]. Schizophrenia and bipolar disorders both share a common trait of autonomic dysregulation, which is purported to be detectable through an assessment of heart rate variability [105,106]. People with schizophrenia showed lower levels of HRV compared to controls [97]. Patients with schizophrenia showed significantly lower high-frequency (HF) and low-frequency (LF) power compared with healthy controls, with a trend towards a higher LF/HF ratio [107]. In people with schizophrenia, illness severity, particularly positive symptoms, is associated with parasympathetic deregulation [97]. In addition, several studies conclude that a reduction in HRV correlates with the severity of this psychiatric disorder [108,109]. The association, however, is not strong, which has been attributed to a variety of factors including the variability between studies in terms of the methods of recording and reporting the data [110]. Administering electroconvulsive shock therapy (ECT) to patients with schizophrenia changes HRV. Following ECT, a shift in autonomic balance can be observed from sympathetic dominance towards increased parasympathetic activity and a state more closely resembling that in healthy controls [107], suggesting that changes in HRV can be used to detect improvements in psychiatric condition. The question of whether psychiatric drugs account for the reduction in HRV has been answered by studies reporting that there is a small and largely non-significant impact of antipsychotic medication on HRV in people with schizophrenia [7].

The data applying AI to HRV in schizophrenia are very limited.

Ksiazek et al. studied 30 individuals diagnosed with schizophrenia or bipolar disorder and 30 control subjects [111]. HRV data was evaluated using multiple machine learning models, including Support Vector Machines, XGBoost, multilayer perceptron, Gated Recurrent Units, and ensemble methods. They concluded that their method achieved a classification accuracy of 83% for the five-fold cross-validation and 80% for the leave-one-out scenario [111]. The combination of patients with schizophrenia and depression limits the extrapolation to only cases of schizophrenia.

Summarizing the data on the different psychological and psychiatric conditions show a range of acuracy, the most common metric reported in most of the studies (Table 5).

## 9. Comments and Challenge

AI—particularly machine learning (ML) approaches considering heart rate variability (HRV)—offers several key advantages for the assessment of psychological and psychiatric conditions. In the context of depression, these advantages have been organized into two tiers, a framework that can be extended to the other conditions discussed herein [112].

The first and more impactful tier includes AI-based modeling, natural language processing (NLP)-driven analysis, and real-time monitoring capabilities. ML models can capture complex, non-linear relationships between HRV features and clinical states, improving the linkage between physiological signals and specific conditions. NLP techniques further enhance this ability by extracting clinically relevant insights from unstructured data, such as patient-reported symptoms or social media content, thereby supporting more nuanced and scalable diagnostic assessments. Real-time monitoring, enabled by wearable devices, allows for continuous and dynamic tracking of physiological signals, offering a substantial advantage over traditional point-in-time assessments.

The second tier includes passive data collection and multimodal data integration. Passive data collection reduces reliance on clinician-administered assessments, enabling scalable and less resource-intensive monitoring. Importantly, HRV data can be integrated with other physiological signals—such as skin temperature, respiratory rate, and galvanic skin response—to create a more comprehensive and robust representation of an individual’s state. This multimodal approach supports more informed and potentially more accurate clinical decision-making by combining independent and complementary data streams.

With respect to model selection, no single approach is universally superior. The evidence suggests that random forest models can achieve a performance comparable to deep learning methods in certain contexts [113]. Moreover, while deep learning is often criticized for its limited interpretability, it can, in some applications, provide levels of interpretability that are comparable to other methods. Model performance is highly dependent on data set characteristics—particularly sample size and subjects’ characteristics, as well as model configuration [113,114].

**Figure 2:**
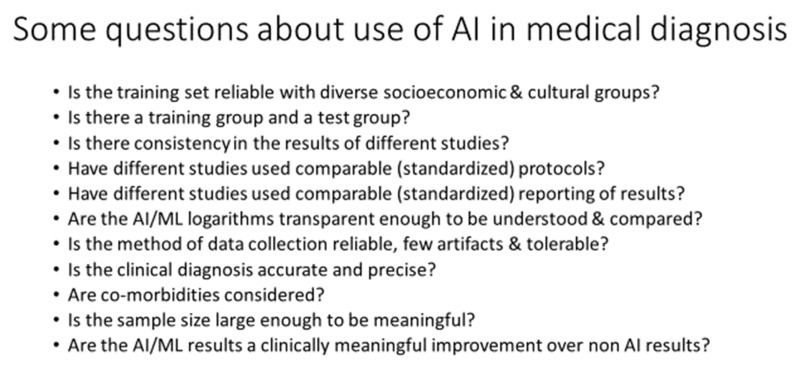
Questions and concerns in the data linking AI or machine learning algorithms in the diagnosis of and when guiding treatment for psychological and psychiatric conditions.

Critiques of the literature on HRV and psychological or psychiatric condition encompass a number of issues (Figure 2).

- Is the training set reliable, including diverse socioeconomic and cultural groups?
- Is there a training group and a test group?
  - Some studies present the results from their original group (training data set) only and do not apply or test their model on a totally different group or population, the so-called test group.
- Is there consistency in the results of different studies?
- Have different studies used comparable (standardized) protocols?
  - The lack of standardized protocols between studies limits, and in some cases precludes, between-study comparisons.
  - There is a need for generally accepted protocols for stress-level annotation and the standardization of HRV metrics.
- Have different studies used a comparable (standardized) reporting of results?
  - Some studies report only sensitivity and specificity, while others report only AUC, and others do not calculate F1.
  - There is a lack of consistent reporting practices.
  - Are the AI/ML logarithms transparent enough to be understood and compared?
  - Concerns remain that the ‘black box’ of machine learning is impenetrable, therefore creating a lack of transparency in understanding how the models are constructed.
  - Differences in AI or ML methodologies between studies create challenges in identifying the best approach (SVM, gradient boost, etc.) to apply and accept into clinical practice.
- Is the method of data collection reliable and tolerable, with few artifacts?
  - The compliance of patients with different psychological or psychiatric conditions is needed for them to wear the recording device.
  - Artifacts that may result from the technology or poor adherence to recording techniques must be managed.
  - There are differences in the mode of data collection—machine or wearables—and the spectrum of different wearables obtain different frequency responses.
  - The system must work in real-world settings and not just in the lab.
  - Are there multi-day/multi-week studies with external validation?
- Can the computational complexity be adapted to lightweight, energy-efficient wearables?
- Is the clinical diagnosis accurate and precise?
  - Are there differences in diagnostic criteria between studies?
- Are co-morbidities considered?
- Is the sample size large enough to be meaningful?
  - The small sample sizes in some studies limit the ability to extrapolate the study.
- Do the AI/ML results show a clinically meaningful improvement over non-AI results?

Bias in AI models used for mental health assessment can arise from multiple sources, including non-representative training data sets, embedded algorithmic assumptions, and unequal access to digital technologies across socioeconomic and cultural groups [112]. A central limitation in the field is the lack of large, diverse, and standardized data sets that enable analyses stratified by key variables, such as age, sex, and socioeconomic status. Without such data sets, the results remain difficult to generalize and compare across studies.

To address this, the field urgently requires widely accepted standards—both for stress annotation protocols and for the calculation and reporting of heart rate variability (HRV) metrics. Standardization is essential not only for reproducibility but also for enabling meaningful comparisons of model performance across studies. Without it, claims of accuracy and clinical utility remain fragmented and difficult to interpret.

The consequences of biased or poorly validated inputs are particularly serious in clinical contexts, where they may contribute to misdiagnosis or unequal care delivery [112]. In addition, many current models are developed under controlled laboratory conditions and fail to account for real-world challenges such as motion artifacts, environmental noise, and variability in user behavior. Bridging this gap requires the development of robust algorithms capable of maintaining performance under degraded signal-to-noise conditions and across shifting data domains.

Future research must also prioritize high-quality prospective studies—ideally conducted over multiple days or weeks—and include rigorous external validation. At the same time, computational methods must be adapted for deployment on lightweight, energy-efficient wearable devices without compromising performance.

Beyond technical challenges, there are important gaps in clinical guidance, regulatory oversight, and professional training related to AI integration in healthcare. These gaps raise concerns about misuse, including the potential replacement of established clinical services in ways that could exacerbate existing health inequalities [115]. Addressing these issues should be a central focus in the design of future studies and implementation strategies.

## 10. Conclusions and Future Directions

The integration of wearable devices with AI has the potential to significantly advance the screening, diagnosis, and management of depression and anxiety [57]. However, realizing this potential requires demonstrating that these technologies are both accurate and clinically meaningful.

Future research should determine whether HRV-derived features can reliably distinguish between different psychological and psychiatric conditions, rather than merely detecting general stress states. This includes more granular investigation of HRV frequency bands and their condition-specific signatures.

There is also a need to develop publicly available, well-annotated multimodal data sets using unified protocols. Such data sets should integrate physiological signals with behavioral and contextual data (e.g., smartphone usage, speech, and text), while ensuring transparency, privacy, and ethical data governance.

In parallel, research should focus on personalized and adaptive modeling approaches that account for individual baseline HRV patterns and inter-individual variability in stress responses. Hybrid systems that combine physiological sensing with passive behavioral data may offer improved accuracy, but must remain interpretable and transparent to clinicians.

Critically, AI-based analyses must demonstrate clear, incremental clinical value beyond traditional HRV metrics alone. Without this added benefit, the justification for the increased complexity is limited. These technologies should ultimately be evaluated based on their ability to support accurate, continuous mental health monitoring and to inform personalized treatment strategies, including both pharmacologic and non-pharmacologic interventions.

## Figures and Tables

**Table 1:** ML models for the detection of stress, Bahameish et al. [46].

Metric	F1 Score
Logistic regression	87.2
Decision tree	87.1
K-nearest neighbor	84.0
Naive Bayes	84.4
Random Forest	89.2
Support vector machine	84.3

**Table 2:** The ability of the algorithm to detect the stressful condition, Bahameish et al. [46].

Metric	Score
F1 Score	65.8%
Accuracy	70.3%
Precision	100%
Recall	49.1%
AUC	53.6%
MCC	64.2%

**Table 3:** Two ML models for the detection of depression ([91]).

	ERTC	SVM
F1 score	89%	75%
Specificity	75%	80%
Accuracy	83%	79%
Precision	83%	73%

**Table 4:** ML models for the detection of depression (Xia et al. [92]).

Metric	F1 Score
Gradient Boosted Machine (GBM)	88.3
LightGBM	84.1
XGBoost	85.9
Linear discriminant analysis	84.7
Logistic regression	83.6
K-nearest neighbor	85.0
Multilayer perception	85.1

**Table 5:** Accuracy of ML algorithms in the diagnosis of certain psychological or psychiatric conditions.

**Mental stress**		
**Author**	**ML algorithm (best one if multiple were used)**	**Accuracy**
He et al. [40]	SVM binary classification SVM multi-class classification	76 79%
Cinaz et al. [38]	SVM, LDA and KNN	71–86%
Fan et al. [41]	SVM	80%
Parent et al. [42]	LR SVM	42% 82%
Giannakckis et al. [43]	RF Pair-wise SVM	75% 84%
Iovino et al. [44]	LDA, SVM, KNN and RF	80%
Castaldo et al. [45]	LDA	94%
Bahameish et al. [46]	RF (test set)	70%
Lei et al. [47]	KNN	93%
Lee et al. [48]	SVM	91%
Huang [49]	RF (one data set) MLP (another data set)	73% 67%
**Anxiety disorders**		
Gu & Hu [64]	SVM LSTM SVM + LSTM	67% 73% 86%
Li et al. [65]	MLP	79%
Handouzi et al.	LSTM	98%
Xia et al. [80]	GBM	83%
**Panic disorders**		
Na et al. [73]	Logistic regression	78%
Oh et al. [74]	Random Forest	71%
Tsai et al. [72]	LSTM RNN	93% 91%
**Depression**		
Kobayashi et al. [85]	SVM	87%
Zhang et al. [86]	Fuzzy-based model	95%
Sun et al. [87]	Logistic regression	79%
Kuang et al. [88]	Bayesian	87%
Kim & Lim [89]	Neurofuzzy network	85%
Byun et al. [90]	SVM	74%
Li et al. [65]		82%
Geng et al. [91]	Ensemble learning decision tree	83%
Xia et al. [92]	Gradient-Boosted Machine	83%

See text for abbreviations.

## Data Availability

The data is available and published in the literature.

## Supplementary Materials

The following supporting information can be downloaded at: https://www.mdpi.com/article/10.3390/bioengineering13050554/s1, Figure S1: Modified from: Page MJ, et al. BMJ 2021;372:n71. doi: 10.1136/bmj.n71.
