# Supplementary material for: Artificial Intelligence in the Assessment of Heart Rate Variability as an Instrument to Understand the Connection Between Psychologic and Psychiatric Conditions and the Heart
Source: Bioengineering (Basel). 2026 May 14;13(5):554. doi: 10.3390/bioengineering13050554 (PMC13203877; doi:10.3390/bioengineering13050554)
Supplement: Supplementary file 1 [file bioengineering-13-00554-s001.zip › bioengineering-4076974-supplementary.pdf]

Artificial intelligence in the assessment of heart rate variability as an instrument to understand the connection of psychologic and psychiatric conditions and the heart

Simon W. Rabkin

The research question was the relationship of heart rate variability, after artificial intelligence or machine learning modeling, to mental stress or anxiety disorders or panic attacks or depression or schizophrenia. This systematic review was conducted according to the Preferred Reporting Items for Systematic Reviews and Meta-Analyses (PRISMA) guidelines. The review protocol was not previously published. A literature search was conducted across in MEDLINE from database inception to October 31, 2025. The search stratergy was Artificial intelligence OR Machine learning AND heart rate variability COMBINED with mental stress OR anxiety disoder OR panic attacks OR depression OR sccizophrenia. The inclusion criteria were all primary research papers published in English that examined the utility of AI, machine learning and HRV data in the identification of the above noted pschological states or psychiatric conditions. The following inclusion criteria were used adult (age ≥ 18) persons undergoing mental stress, or diagnosed with anxiety disorders, panic disorders, depression or schizophrenia. Exclusion criteria included non-human studies, pediatric age groups as well as editorials, commentaries, conference abstracts, reviews, or non-English studies so that the methods and results could be reviewed in detail.

The flow diagrma is as follows

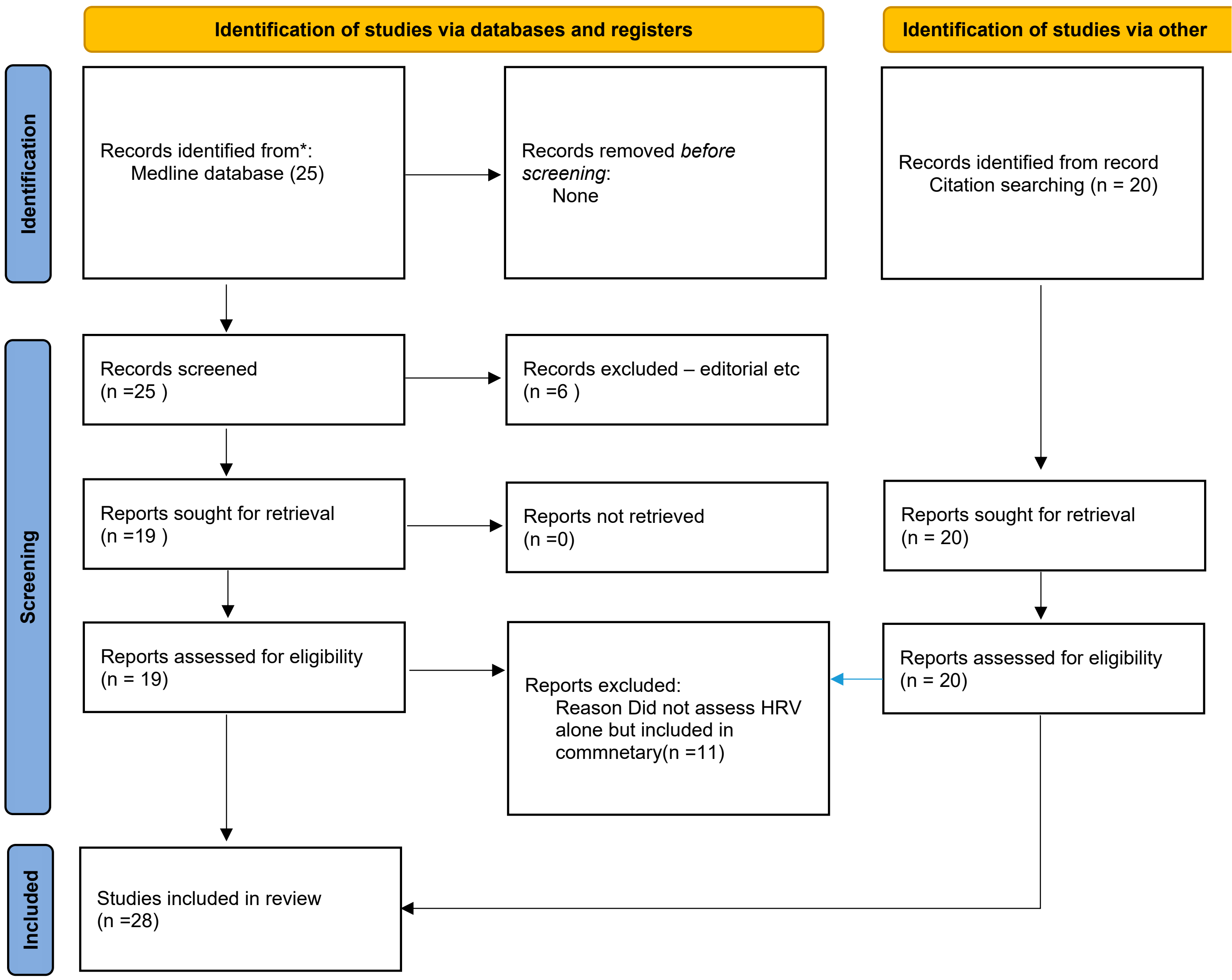

*

Modified from : Page MJ, et al. BMJ 2021;372:n71. doi: 10.1136/bmj.n71.
